# Advances in transcatheter mitral and tricuspid therapies

**DOI:** 10.1186/s12872-019-01312-3

**Published:** 2020-01-07

**Authors:** Pavel Overtchouk, Nicolo Piazza, Juan Granada, Osama Soliman, Bernard Prendergast, Thomas Modine

**Affiliations:** 1 Department of Cardiology, University Hospital of Bern, Bern, Switzerland; 2 Interventional cardiology, McGill University Health Centre, 1001 Boulevard Décarie, Montréal, QC H4A 3J1 Canada; 3 Cardiovascular Research Foundation, Columbia University Medical Center, CRF Skirball Center for Innovation, New York, USA; 4 Thoraxcenter, Erasmus University Medical Center Rotterdam, Medical Director Structural Heart & Heart Failure Research, Cardialysis, Rotterdam, Netherlands; 5 Department of Cardiology, University Hospital of Antwerp, Antwerp, Belgium; 6 Department of Cardiology, St Thomas’ Hospital, London, UK; 7 Cardiology and Cardiovascular Surgery Department, Heart Valve Center, Institut Cœur Poumon CHU de Lille, 2 Av Oscar Lambret, 59037 Lille, France; 8 Jioa Tong university, Shanghai, China

**Keywords:** TMVI, TMVR, TTVI, Transcatheter mitral therapy, Transcatheter tricuspid therapy, Mitral repair, Mitral replacement, Tricuspid repair, Tricuspid replacement

## Abstract

**Background:**

While rheumatic mitral stenosis has been effectively treated percutaneously for more than 20 years, mitral and tricuspid regurgitation treatment appear as a contemporary unmet need. The advent of transcatheter therapies offer new treatment options to often elderly and frail patients at high risk for open surgery. We aimed at providing an updated review of fast-growing domain of transcatheter mitral and tricuspid technology.

**Main body:**

We reviewed the existing literature on mitral and tricuspid transcatheter therapies. Mitraclip is becoming an established therapy for secondary mitral regurgitation in selected patients with disproportionately severe regurgitation associated with moderate left ventricle dysfunction. Evidence is less convincing for primary mitral regurgitation. Transcatheter mitral valve replacement is a promising emerging alternative to transcatheter repair, for secondary as well as primary mitral regurgitation. But further development is needed to improve delivery. Transcatheter tricuspid intervention arrives late after similar technologies have been developed for aortic and mitral valves and is currently at its infancy. This is likely due in part to previously under-recognized impact of tricuspid regurgitation on patient outcomes. Edge-to-edge repair is the most advanced transcatheter solution in development. Data on tricuspid annuloplasty and replacement is limited, and more research is warranted.

**Conclusion:**

The future appears bright for transcatheter mitral therapies, albeit their place in clinical practice is yet to be clearly defined. Tricuspid transcatheter therapies might address the unmet need of tricuspid regurgitation treatment.

## Background

Valvular heart disease (VHD) is one the most frequent pathologies treated with cardiac surgery in western countries. Interventional and surgical treatment remains the cornerstone of VHD treatment. Mitral stenosis is less frequent than mitral regurgitation (MR) in developed countries. MR was reported to be present in > 1% of Western type population after 70 years old and associated with an increased mortality [1]. Mitral valve surgery represents one tenth of all surgical procedures but this remains insufficient to address the challenge of interventional treatment of mitral valve disease [2–5]. The incidence of significant tricuspid regurgitation (TR) after 70 years old is > 5% of the population. Moderate or severe TR has been incriminated to be responsible with long-term mortality, but is infrequently treated with surgery or percutaneous intervention [6–8]. It represents the most common pathology of the tricuspid valve, and TR is functional (or secondary to conditions responsible of right ventricle or atrium dilatation) in an overwhelming 90–95% of cases as opposed to the primary aetiology (e.g. trauma, radiation, endocarditis) [9]. The perceived invasiveness of open surgery and an insufficient consideration of MR and TR to genuinely impact patient survival and symptoms might be reasons for their insufficient interventional treatment, hence they are possible targets for research.

We aimed at providing a narrative review of the published literature on transcatheter mitral and tricuspid interventions.

## Main text

### Transcatheter mitral valve therapies

#### Percutaneous mitral commissurotomy for rheumatic mitral stenosis

The idea of treating the mitral valve disease percutaneously dates back to the percutaneous mitral commissurotomy (PMC) with the Inoué balloon for severe symptomatic rheumatic mitral stenosis [10]. PMC is effective, has a lower procedural morbidity and mortality than open surgery, and reserves the possibility to re-intervene in case of recurrence [11]. But eligibility criteria for PMC are strict and if not present open surgery is to be favoured **(**Table 1**)** [11].

**Table 1:** Characteristics of unfavourable anatomy for percutaneous mitral commissurotomy

Wilkins score > 8 (echocardiographic): immobile leaflets, thick mitral leaflets and sub-valvular apparatus, extensive calcification	
Cormier group 3 (imaging): calcification of mitral valve of any extent as assessed by fluoroscopy	
Very small mitral valve area	
Non-rheumatic mitral stenosis	
Severe tricuspid regurgitation	
Left atrial thrombus	
Concomitant indications for heart surgery such as coronary artergy bypass graft	

#### Edge-to-edge transcatheter mitral valve plasty

Devices designed for transcatheter treatment of MR have often been inspired by surgical techniques. Hence, transcatheter-based devices can be organised into replacement and repair techniques [12]. The MitraClip (MC) device (Abbott Vascular, Santa Clara, CA, USA) resulted from the adaptation of the surgical Alfieri valvuloplasty at the beginning of the century [13–15]. The clipping device has since been through several iterations (NT, NTR, XTR) which increased its size, grasping and maneuverability. Made of cobalt-chromium covered with polyester, the implant has two arms able to grasp the two leaflets and is delivered transseptally. It reduces the mitral regurgitation orifice by “stitching” them together. Multiple clips can be positioned to maximize results.

The first trial having investigated the efficacy and safety of the MC device was the EVEREST II randomized controlled trial. Were compared the MC to surgery for the treatment of primary or secondary MR [15]. EVEREST II found that MC was inferior to open mitral surgery regarding the primary efficacy composite endpoint of freedom from death, surgery for mitral-valve dysfunction and MR grade ≥ 3+ at 1 year. The primary endpoint was driven by the need for complementary mitral surgery which was higher in the MCgroup. However, subgroup analysis suggested that MC could be a better match for functional MR [15]. At 5 years mortality was numerically higher in the MC group. Furthermore, more than one third of patients either had persistent or recurrent MR grade ≥ 3+ or mitral surgery. Nevertheless, interventional guidelines recommendeds transcatheter edge-to-edge repair for patients with primary [16] and secondary MR at high risk for surgery [11].

More recently, were published two landmark randomized trials that compared MitraClip to medical therapy alone in patients with severe secondary MR, moderate left ventricle dysfunction, and suitable anatomy for MC implantation: COAPT and MITRA-FR trials. In the COAPT trial, there was a benefit from the MC at 2 years in the form of reduced long-term mortality) and rehospitalization for heart failure. In the MITRA-FR trial, the rate of mortality at 1 year was similar for the intervention and control groups, respectively; the rate of rehospitalization for heart failure was also similar for the intervention and control groups, respectively [17, 18]. One of the major challenges in both trials was the selection of patients for inclusion. Both struggled with inclusions since it took 78 north American centers to include 614 patients in 4.5 years in COAPT (1.7 patients per center per year), and 3.4 years to 37 French centers to include 304 patients in MITRA-FR (2.4 patients per center per year). In MITRA-FR one third of the eligible patients were excluded after enrollment, essentially after echocardiographic eligibility assessment. In COAPT 58% of patient were excluded after enrollment (more patients were excluded than included), again essentially after echocardiographic assessment.

Considering both trials together, one might postulate that MitraClip might benefit very selected in whom the functional MR is thought to be responsible for a worsening left ventricle function, and not solely the result of a progressive enlargement of the left ventricle and atrium. Some authors proposed the concept of “tertiary” MR to describe these patients, in whom MR severity is excessive with regard to a moderate left ventricle dysfunction and dilatation [19]. Consecutively, the upcoming international guidelines are likely to adopt a COAPT-like approach and propose criteria for MC aiming at patients with a “tertiary” functional MR rather than the currently recommended compassionate approach [11]: moderate or severe, LVEF 20–50% and left ventricle end-systolic diameter ≤ 70 mm with symptoms despite maximally tolerated medical therapy. The results of the RESHAPE-HF2 (NCT02444338) and MATTERHORN trial (NCT02371512) trials might provide more insight on the prospect of a future for the MitraClip device for the treatment of functional MR. It is worth noting that Mitraclip repair is guided by transoesophageal echocardiography, thus the feasibility as well as the result of the procedure are dependent on the echogenicity of the patient and the skills of the interventional imager.

**Fig. 1:**
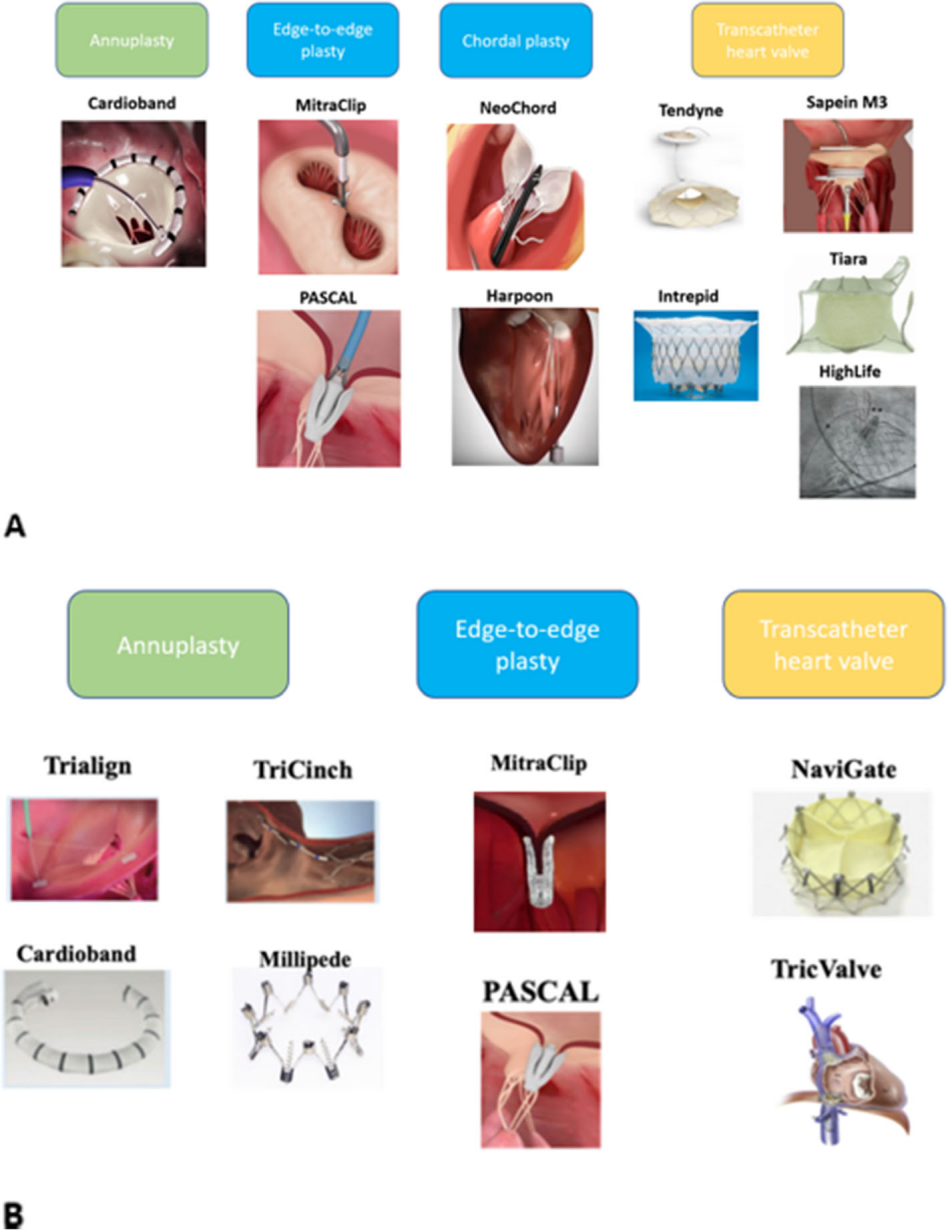
Transcatheter mitral (**a**) and tricuspid (**b**) valve intervention devices with reported clinical use (original image)

The Pascal™ (Edwards Lifesciences) edge-to-edge mitral plasty device is a repositionable and recapturable system. The device has two paddle-shaped grasping arms that are independently closable (clasps) as well as a central spacer that is intended to fill the regurgitant jet area. After a favourable initial experience for compassionate use, PASCAL has been demonstrated to be safe and effective enough for clinical use in the CLASP prospective multicenter cohort study. 62 patients have been included in the CLASP cohort, with both degenerative and functional MR, and observed a low 1.6% cardiovascular mortality, without any stroke events and a 98% rate of < 3+ MR at 30 days [20, 21] (Table 2**,** Fig. 1).

**Table 2:** Short term (in-hospital or 30-days) outcomes of TMVI devices

	Technical success*	Mortality	MR 2+ or more
Transcatheter repair			
MitraClip [22]	178/178	2/178	2/178
Pascal [20]	18/23	3/23	7/19
Cardioband [23, 24]	43/60	3/60	18/57
NeoChord [25, 26]	89/93	1/92	30/92
Harpoon [27]	28/30	0	3/27
Transcatheter replacement			
Tendyne [28]	97/100	6/100	21/94
Intrepid [29]	48/50	7/50	0/42
Sapien M3 [30]	9/10	0	1/10
Tiara [31]	17/17	1/17	–
HighLife [32]	9/11	3/11	–

#### Transcatheter annuloplasty and chordal plasty

More recent are transcatheter annuloplasty (such as CardioBand™, Edwards Lifesciences) and chordal replacement (such as NeoChord™ NeoChord, Inc.; or Harpoon™ Edwards Lifesciences) systems [23, 25]. The Cardioband is a flexible implant with multiple anchors which are attached to the annulus, and once they are all fixed, tensions can be applied reducing the dilated annulus to a physiological size. The device is delivered through a flexible catheter transseptally. A recent publication reported 1-year outcomes after Cardioband implantation in 60 patients with moderate or severe secondary MR treated in 11 European institutions. While 30-day mortality was below 5%, the MVARC-defined procedural success was only of 68%, more than mild MR remained after the procedure in 30% of patients, reintervention was frequent during the 1-year follow-up [23, 24]. These data were consistent of those published on short term outcomes. Overall, while the safety of the Cardioband device delivered through transfemoral access seems acceptable, its reported efficacy regarding durable MR correction remains insufficient. NeoChord and Harpoon are neo-chordal technologies which attach synthetic new chords to mitral leaflets and myocardium of the left ventricle. Both are logically transapically implanted. Observational data reported low 30-day mortality (< 2%) but remaining MR grade ≥ 2 in 10 to 35% of patients after the procedure [25–27].

#### Limitations of transcatheter plasty and the case of transcatheter replacement

Despite extensive preoperative echocardiographic screening in the COAPT trial, more than one clip was necessary in > 60% of patients (≥ three clips in 8% of cases) to achieve satisfactory reduction of MR [17]. In the EVEREST II trial, 5-year data reported that patients included in the surgical group which comprised essentially patients with mitral repair, required mitral re-do surgery during the 5 years of follow-up in 9% of cases. This is higher than previously suggested in the observational studies [33–35]. Observational studies tend to underestimate outcomes due to lack of follow-up and underreporting. Furthermore, randomized trial data on the impossibility of repair and switching to replacement, as well as the need of reoperation after mitral repair is scarce. Thus, this new estimation of 9% reoperation 5 years after repair in EVEREST II is to consider for the ongoing debate of mitral repair versus replacement [12]. Indeed, surgical mitral bioprosthetic replacement seems to yield a reoperation rate at around 2% which could be transposable to transcatheter mitral replacement [36]. Combining transcatheter repair techniques has been proposed to mitigate the lack of efficacy on MR resolution; however, this poses the question of increased complication risk and cost [37, 38]. Perhaps valve replacement could provide a better option.

#### Transcatheter mitral valve replacement

Mitral repair is favored over replacement for open surgical treatment of MR in the international guidelines [11]. However, this recommendation is based on observational data. Recent randomized data showed that replacement nearly eliminates the risk of long-term recurrence of moderate or severe MR at 2 years (58.8% after repair vs 3.8% after replacement) [39]. By avoiding the morbidity of open mitral surgery and effectively preventing recurrence of MR, transcatheter mitral valve replacement (TMVR) could provide the best option [12].

The first-in-human TMVR was performed in 2012with the CardiAQ valve (Edwards Lifesciences) [40]. More recently were published two TMVR feasibility studies. One of them included patients treated with the Tendyne (Abbott Structural Heart) device, while the other with the the Intrepid (Medtronic) transcatheter heart valve. The studies included patients at very high surgical risk and both devices were implanted transapically The 30-day mortality rate was 14% with the Intrepid device and 6% in the Tendyne study (26% at 1 year). However, it is important to notice that the complete correction of significant MR was constant in both studies [28, 29, 41]. Following these favorable results, the first trials aiming at comparing TMVR with to open surgery are already underway. In the he APOLLO trial (NCT03242642) patients in the TMVR group will be treated with the Tendyne device, while in the SUMMIT trial the TMVR device will be the Intrepid (NCT03433274).

The recent report of a successful first-in-man implantation of the Abbott CEPHEA™ is notable because of the lack of transseptally implantable transcatheter mitral valves [42]. The CardioValve Mitral Tech™ is another example of this promising technology. Both devices are delivered transseptally, which illustrates the urge to develop transcatheter heart valves (THV) that will be delivered through a less invasive approach than transapical. Numerous feasibility and safety single-arm studies with other transapical and transseptal TMVR devices are underway. It is the case for Edwards EVOQUE™ (NCT02718001), Neovasc Tiara™ (NCT02276547) and the HighLife™ (NCT02974881).

The first TMVR systems use 32- to 45-F transapical delivery catheters which are incompatible with percutaneous accesses. Thus, adaptation of the technology for transseptal implantation is a major engineering challenge. The trileaflet Sapien 3 29 mm device used in for transcatheter aortic replacement was previously used transseptally for valve in ring and valve in valve mitral replacement. Hence, its manufacturer logically adapted the device for the native mitral valve (renaming the device Sapien M3), taking advantage of a decade-long experience in transcatheter aortic interventions and its already developed delivery system. Given the absence of a solid armature for device anchoring the Sapien M3 THV uses an expandable polytetrafluoroethylene–covered nitinol “dock” which allows its stabilizing by interacting with the mitral subvalvular apparatus. Webb et al. recently published their experience in a short cohort of 10 patients with primary and/or secondary MR and reported a technical success rate of 90%, without stroke or death at 30-days.

It is worth citing the reported experience of TMVR using TAVI devices (essentially Edwards Sapien family, and infrequently Boston Lotus). The need for a solid armature for the THV to be implanted restricted the experience to “Valve in valve” (VIV) for degenerated mitral bioprostheses, “valve in ring” (VIR) for mitral disease after annuloplasty ring surgery and “valve in mitro-annular calcification” (VIMAC). While results were satisfactory for VIV TMVR with an approximative 95% technical success rate, results of VIR and VIMAC are less appealing with technical success rates of 80 and 62% in observational cohorts respectively [43]. 30-day mortality follows a similar pattern: 6, 10 and 35% for VIV, VIR and VIMAC respectively. The results of VIMAC underscored the limited solutions for patients with severe mitral valve disease at high risk for surgery and mitro-annular calcification. But, recent data on VIMAC TMVR using THVs designed for the mitral valve, such as the Tendyne™, have been reported to yield encouraging results in compassionate cases and is under clinical investigation.

Furthermore, TMVR carries risks [44]. Although less frequent with THVs dedicated to the mitral valve (< 1%), left ventricle outflow tract obstruction (LVOTO) has been reported in 1/8 patients when aortic THVs devices were used for TMVR [28–30, 45]. LVOTO is defined by an increase of 10 mmHg or more of the transaortic gradient [46] and associated with higher mortality [43, 47–49]. New methods have been proposed as bailout procedures when operators confronted LVOTO. Septal alcohol ablation allowed rapid haemodynamic improvement in most of patients in a short case series of 6 patients [50]. Rescue laceration of the anterior mitral leaflet (LAMPOON technique) is another alternative [51, 52]. In a more preventive approach, Wang et al. reported an increase of the neo-LVOT surface area after pre-emptive alcohol septal ablation, but at the cost of an increased risk of major conduction disturbances requiring a permanent pacemaker implantation [53].

#### The challenge of patient selection

Eligibility of patients to transcatheter mitral interventions is often reduced to inoperable or very high patients for open surgery. However, given the financial stakes of percutaneous mitral intervention, industry has been heavily investing in the development of such technologies [54]. Hence it is likely that new percutaneous solutions will become mainstream within the next 5 years. Then will remain the problem of the cost of those devices.

However, on the opposite of the aortic valve which function largely stands for itself, the function of the mitral valve is intertwined with the anatomy and function of the left ventricle as well as the left atrium. Mechanisms of mitral regurgitation are often numerous and interconnected, and the correction of one mechanism might result in the correction or worsening of another. For instance, the reduction of mitral regurgitation might result in reduction of left ventricle volume (given remodeling is possible) and hence of the mitral annulus dilatation, overall resulting in further reduction of mitral regurgitation. On the other hand, not respecting the mitral sub-valvular apparatus during surgical mitral replacement is deleterious for left ventricle geometry and function [55]. Thus, predicting the effect of a given intervention requires considering its impact on the left heart rather than the mitral valve alone.

Besides, on the contrary of surgical repair involving a quasi-mandatory association of several repair techniques (annuloplasty, leaflet plasty, chordal plasty), transcatheter repair devices are very specialized and allow the treatment of a single mechanism though to be dominant. Attested by the extensive list of device contra-indications and incompatibilities, room for adaptation to an individual patient anatomy is very limited by device size as well as device and delivery system conformation.

Targeting patients with functional MR who could benefit from Mitraclip has been improved by the publication of COAPT and MITRA-FR trials. However, such data is scarce for transcatheter annuloplasty devices and TMVR. Anatomical compatibility with TMVI devices is of central importance. TMVI require extensive pre-operative feasibility screening including transesophageal echocardiography and MSCT with 3-dimensional reconstructions, to verify mitral anatomy compatibility and pathway patency. Finally, the possibility to re-intervene could be crucial given that device durability remains uncertain for the recent TMVI and TMVR technologies. Transcatheter repair devices can be combined (i.e. edge-to-edge with annuloplasty), however subsequent transcatheter replacement would become impossible, while valve-in-valve transcatheter replacement remains possible.

As of now, open surgical correction of several coexisting valvulopathies yields the most durable results given the possibility to efficiently treat them during the same intervention. Thus, whenever possible, surgery should be favoured in operable patients with coexisting mitral and aortic or tricuspid valve disease [8, 56]. Despite the absence of robust data, it is largely admitted that in cases of inoperable patients with severe mitral regurgitation and secondary severe tricuspid regurgitation, a single intervention on the mitral valve should be considered first since its correction could result in a significant reduction of the secondary tricuspid regurgitation. If the impact of the mitral intervention has been insufficient and symptoms persist a complementary tricuspid intervention can be discussed [57].

### Transcatheter tricuspid valve therapies

The tricuspid valve has been traditionally dubbed the “forgotten valve”. The heterogeneity of etiologies associated with TR renders the evaluation of its impact and the impact of its treatment difficult to estimate. Severe primary TR is a clear indication for surgery but constitutes less than 10% of TR cases. Despite functional TR being very frequently observed on echocardiography (> 50%), it is often only considered for surgical treatment when there is a concurrent indication for left heart surgery [6, 11, 58]. Although without treatment, TR may progressively deteriorate, leading to worse symptoms, biventricular heart failure and death. In a large retrospective analysis of 5223 patients, Nath et al. showed that moderate and severe TR is associated with worse survival even when adjusted for pulmonary artery systolic pressure (PASP), left ventricular ejection fraction (LVEF), RV size and function [8].

However, evidence that surgical correction of an isolated TR improves survival or symptoms is lacking. Based on few observational studies international guidelines recommend surgical annuloplasty of non-severe TR with of annulus dilatation ≥40 mm or > 21 mm/m^2^ by 2D echocardiography and valve repair or replacement for severe TR [56]. Tricuspid valve surgery for secondary symptomatic TR is often performed in small cohorts of high risk patients, with previous left-heart surgery, in the form repair or replacement. This yields a high short term mortality, between 5 and 15% [59–62]. A recent study by Axtell et showed no survival benefit of surgery compared to medical therapy in a large cohort of 3276 patients. No difference was identified between repair and replacement either [63]. However, one of the lessons learned from COAPT and MITRA-FR trials is that a tailored approach could be very successful [19]. Furthermore, previous studies suggested that tricuspid annuloplasty allowed right ventricle recovery, reduces dyspnoea and congestive heart failure [56, 64, 65].

Transcatheter tricuspid valve intervention (TTVI) techniques avoid open surgical morbidity generating conditions such as cardio-pulmonary bypass, sternotomy and intubation, that might improve peri-operative survival [12]. Research and industry are currently very active in this domain [66]. Reported results of TTVI devices are presented in Table 3.

**Table 3:** Short term (in-hospital or 30-days) outcomes of TTVI devices

	Trial/study	Technical success^a^	Mortality	TR volume reduction (mL)
TriAlign	SCOUT I [67] NCT02574650 SCOUT II is enrolling	12/15	0	−2.7 ± 39.5
TriCinch	Giannini and Colombo [66] PREVENT ongoing NCT03632967	20/24	–	–
Cardioband	TRI-REPAIR [68] TriBAND ongoing NCT03779490	28/30	2/30	−35.6 ± 35.3
MitraClip and TriClip	Nickenig et al. [69] TRILUMINATE NCT03227757 [70]	6/64 10/85	3/64 0	−26.4 ± 7.8 − 18.6 ± 21.2
FORMA	Perlman and Dvir [71–73] SPACER enrolling NCT02787408	–	2/47	–

^a^ no standardized definition for “technical success” for TTVI

#### Transcatheter edge-to-edge and spacer tricuspid technology

As for transcatheter mitral valve therapies, percutaneous techniques for tricuspid valve intervention were often inspired by surgical techniques. The Alfieri-styled edge-to-edge surgery has been proposed for the tricuspid valve as well as mitral [74]. A decade long experience with the MitraClip in the mitral position prompted numerous operators to attempt TR correction using the MitraClip in tricuspid position. The best results appear to occur by attaching the anterior and/or posterior leaflet to the septal leaflet, which can also reduce annular dimensions. Clipping the anterior and posterior leaflets is generally not advised because it may distort the valve and worsen TR. More recently the “TriClip” was introduced as a transcatheter tricuspid valve repair system. It is essentially a modification of the MitraClip NT’ percutaneous delivery system (both owned by Abbott) and was investigated in the TRILUMINATE study (NCT03227757).

The PASCAL device has also been successfully adapted from mitral to tricuspid use in one case report [75]. However, data regarding its safety and efficacy are lacking. The Forma device (Edwards Lifesciences) has been advocated to reduce TR by creating a new surface for coaptation for tricuspid leaflets. It consists of a foam-filled spacer, available in 12 and 15 mm both with a length of 42 mm, that is inserted via the subclavian or the axillary vein, placed in the regurgitant orifice and anchored in the RV apex [76]. The first-in-man experience with 7 patients and a feasibility study that included 16 patients that showed reduction in TR. More data is awaited from the ongoing SPACER study (NCT02787408).

#### Transcatheter annuloplasty techniques

Nowadays abandoned, the Kay suture annuloplasty consists of excluding the posterior valve leaflet for “bicuspidation” of the tricuspid valve by tightening a suture from the anteroposterior commissure to the posterior-septal commissure (“Kay technique”) [77]. Another technique uses two parallel lines of running sutures starting at posterior-septal commissure at the annulus level with a stich to the fibrous trigone to narrow the tricuspid annulus (“De Vega technique”) [78]. Those surgical annuloplasty techniques have been imitated by the TriAlign and TriCinch devices [79, 80]. The Trialign device is a transcatheter suture annuloplasty technique performed transjugularly. An insulated radiofrequency wire is advanced into the right ventricle to then retrogradely cross the tricuspid annulus tissue. Thereafter, two pledgets are placed at the posteroseptal as well as the anteroposterior commissures, which are then cinched to obliterate the posterior tricuspid leaflet, yielding a “bicuspidisation” of the tricuspid valve [79, 81].

The TriCinch device is delivered through the femoral vein. It presents an epicardial coil with two haemostasis seals implanted in the mid-anterior part of the tricuspid annulus, a nitinol stent connected to the coil through a Dacron band, is placed in the inferior vena cava (IVC), to maintain tension applied to the annulus. TriCinch clinical use has only been described in small case series [82–84]. The ring annuloplasty technique is the currently preferred by most teams and uses rigid or semi-rigid rings, planar or non-planar to fit the tricuspid anatomy [85, 86]. The transcatheter equivalent ring annuloplasty can be performed with the Cardioband (also used for mitral annuloplasty) or Millipede devices.

#### Transcatheter tricuspid valve replacement

Only bioprosthetic valves can be implanted percutaneously. Existing THVs usually used for pulmonary stenosis (Melody) and aortic stenosis (Sapien family) have been used for degenerated tricuspid bioprostheses for many years, with a 30-day mortality of 3.2% in the largest registry to date [87]. However, in the absence of solid armature, these devices cannot be used for native tricuspid valves. Existing dedicated TTVR devices are intended to be implanted in a orthotopic or heterotopic positions. The proximity of conduction pathways render complete atrioventricular block one of the expected main complications of TTVR.

The NAVIGATE (NaviGate Cardiac Structures) transcatheter heart valve (THV) is a self-expanding bioprosthesis for orthotopic tricuspid valve replacement (TTVR) that consists of three xenogeneic pericardial leaflets seated in a tapered nitinol stent with atrial winglets and ventricular graspers for anchoring the tricuspid annulus and leaflets without protruding into adjacent chambers. NAVIGATE is available in four sizes intended for TA diameters ranging from 36 mm to 52 mm. A 42 Fr introducer sheath is used to deliver the valve through a transjugular pathway (or through transatrial minimally invasive right thoracotomy surgical approach). The NAVIGATE is currently the only clinically available TTVR device. The delivery system features two degrees of motion at the tip and allows for a 90° angulation [88, 89]. As of yet, only two short case series have been published [88, 90]. Hence clinical implementation remains at its infancy but has interesting potential. Nevertheless, design improvement needs to reduce sheath size and deliverability.

TricValve is in fact a set of two self-expandable heterotopic THVs with each having nitinol frames, and different designs because intended to be deployed into superior and inferior vena cava respectively, at cavo-atrial inflow. TricValve does not require a pre-stenting of caval veins and available sizes from 28 mm to 43 mm. Few patients have so far been reported to have received TricValve, and since it does not treat the TR per se, its intended use beyond compassionate for symptomatic relief is uncertain [91].

Other devices include TriSol (TriSol Medical), Lux (Jenscare Biotechnology), TRiCares (TRiCares SAS, Paris, France) TTVR devices which are yet to be used in clinical setting. Both devices are orthotopic and self-expanding with bovine pericardial tissue mounted on a nitinol stent frame. The TriSol holds a single bovine pericardial structure with a single dome-shaped leaflet which is attached in two opposite central commissures to create a bileaflet valve. The xenograft is mounted on a self-expanding conical nitinol stent featuring a ventricular skirt of porcine pericardium and an atrial polyester skirt. Anchoring is performed through axial force to reduce the risk of conduction disturbance. The resulting prosthesis is retrievable and repositionable. Its 30 Fr delivery system is intended to be used through a jugular vein access to allow an implantation alignment with the tricuspid valve [92]. The clinical applicability of these devices is yet to be investigated.

## Conclusions

Transcatheter mitral valve therapy is nowadays an established solution for high surgical risk patients with mitral regurgitation and those suffering from rheumatic mitral stenosis. Benefit of transcatheter mitral edge-to-edge repair in secondary mitral regurgitation has been recently established in selected patients. However, transcatheter repair as well as replacement are yet to be proven effective for primary mitral regurgitation. Nevertheless, numerous ongoing trials are promised to shed light on the how much of the promises of those technologies will be met with reality. And if benefit is proven, additional research will be needed to establish whether mitral repair and replacement should be opposed or used in complementarity, notably with regards to device compatibility with the anatomy of individual patients. Transcatheter repair as compared to surgical repair, is often limited by the specialisation of the device which will treat a single of often associated mechanisms of mitral regurgitation. Transcatheter replacement requires larger delivery systems, comes with a limited number of device sizes and conveys the risk of left ventricle outflow tract obstruction. Adaptation of a device comes with major financial constraints, hence in the future some patients with infrequent anatomy could still only be served with open surgery.

Clinical implementation of transcatheter tricuspid therapies is still at its infancy. The development of the field has likely been delayed by a lack of recognized impact of tricuspid regurgitation on symptoms and prognosis. However, it now constitutes a promising alternative for patients with isolated secondary tricuspid regurgitation to a possibly morbid open surgery. But all the steps of development of a new valve technology (including safety, efficacy, optimal patient selection) remain to be followed for transcatheter tricuspid interventions.

Given the complexity of mitral and tricuspid valve disease and the increasingly large armamentarium to treat them, Heart Team discussion remains the main guarantor that every patient is offered the optimal solution for her/him based on contemporary evidence-based argumentation.

## Data Availability

Review type of article. No dataset was used.

## Abbreviations

LVOT: Left ventricle outflow tract
MR: Mitral regurgitation
THV: Transcatheter heart valve
TMVI: Transcatheter mitral valve intervention
TMVR: Transcatheter mitral valve replacement
TR: Tricuspid regurgitation
TTVR: Transcatheter tricuspid valve replacement
